# Coupling of serum CK20 and hyper-methylated *CLIP4* as promising biomarker for colorectal cancer diagnosis: from bioinformatics screening to clinical validation

**DOI:** 10.18632/aging.203804

**Published:** 2021-12-29

**Authors:** Zhongjian Liu, Hui Tang, Wen Zhang, Jinli Wang, Lilan Wan, Xisha Li, Yuping Ji, Na Kong, Yanfang Zhang, Jiangang Wang, Zhang Fan, Qiang Guo

**Affiliations:** 1 Department of Gastroenterology, The First People’s Hospital of Yunnan Province, Kunming, China; 2 The Affiliated Hospital of Kunming University of Science and Technology, Kunming, China; 3 Department of Gastroenterology, The Third People’s Hospital of Yunnan Province, Kunming, China

**Keywords:** colorectal cancer, serum diagnostic biomarkers, DNA methylation, CK20, *CLIP4*

## Abstract

Colorectal cancer (CRC) is one of the most common and lethal malignancies. The identification of minimally invasive and precise biomarkers is an urgent need for the early diagnosis of CRC. Through bioinformatics analysis of 395 CRC tissues and 63 CRC cell lines, CK18, CK20, de-methylated *HPDL* and hyper-methylated *CLIP4* were identified as candidate serum biomarkers. Then, a training cohort consisting of 60 CRC, 30 colorectal adenomas (CA) and 33 healthy controls and a validation cohort consisting of 60 CRC, 30 CA and 30 healthy controls were enrolled. In the training cohort, enzyme-linked immunosorbent assay (ELISA) showed that CK18 and CK20 were all significantly higher in CRC and CA. CK18 diagnosed CRC with 46.67% sensitivity and 87.3% specificity; CK20 diagnosed CRC with 28.33% sensitivity and 90.47% specificity. Methylation-specific PCR (MSP) indicated that de-methylated *HPDL* and hyper-methylated *CLIP4* were significantly detected in CRC and CA. De-methylated *HPDL* diagnosed CRC with 36.67% sensitivity and 93.65% specificity and hyper-methylated *CLIP4* with 73.33% sensitivity and 84.13% specificity. Random combined analysis suggested that CK20/hyper-methylated *CLIP4* diagnosed CRC with 91.67% sensitivity and 82.54% specificity. In the validation cohort, CK20 diagnosed CRC with 36.7% sensitivity and 88.3% specificity and hyper-methylated *CLIP4* with 80% sensitivity and 85% specificity. CK20/hyper-methylated *CLIP4* diagnosed CRC with 95% sensitivity and 81.7% specificity. Compared with serum biomarkers reported before, CK20/hyper-methylated *CLIP4* possessed the potential to be a new effective and precise diagnostic biomarker for CRC.

## INTRODUCTION

Colorectal cancer (CRC) is one of the most common malignancies worldwide and has high mortality rates. In recent years, the incidence of CRC has risen. CRC has become the third most common cancer among males and the second most common cancer among females [1]. CRC development is a complex multistep process that involves a gradual progression from adenomatous polyps to adenomas, and then to malignant carcinomas [2]. From a clinical perspective, CRC is difficult to diagnose early, as patients do not present with symptoms such as colorectal bleeding or anemia until later stages, and the survival rate decreases as the stage of diagnosis increases. Therefore, early detection and rapid diagnosis are important for CRC screening and treatment. Blood serum contains a certain amount of secretory proteins and cell-free DNA (cfDNA) derived from all cells in the body and could be a useful material for screening CRC.

Currently, several serum markers such as carcinoembryonic antigen (CEA), carbohydrate antigen 19-9 (CA199), carbohydrate antigen 125 (CA125), carbohydrate antigen 242 (CA242) and alpha fetoprotein (AFP) have been applied for diagnosing and monitoring CRC in the clinic [3, 4]. These biomarkers achieved 10.39~46.59% sensitivity and 80~95% specificity in diagnosing CRC [5, 6].

Aberrant DNA methylation changes have previously been shown to be an early event in the development of CRC [7] can be detected in cfDNA, making it an ideal and useful biomarker for the early detection of CRC [8, 9]. Currently, various tumor suppressor genes have emerged as potential blood-based methylation markers for CRC including *APC, MGMT, hMLH1, HLTF, ALX4, NGFR, TMEFF2, NEUROG1, SERP2, VIM, RASSF2A, WIF1, RUNX3* and *SEPT9* with sensitivities spanning from 34% to 90% and specificities ranging from 69% to 100% [10, 11].

With the vast amounts of CRC transcriptomics and DNA methylomics data that are continuously generated and easily accessed from published sources, it is possible to use bioinformatics to screen biomarkers for CRC diagnosis, specifically and systematically. In this study, The Cancer Genome Atlas (TCGA), Genotype-Tissue Expression (GTEx) [12], Cancer Cell Line Encyclopedia (CCLE) [13], Gene Expression Profiling Interactive (GEPIA) [14], Human Protein Atlas (HPA) [15], UCSC [16], UALCN [17] and MEXPRESS [18] were used to screen specific secretory protein-encoding genes, de-methylated overexpressed genes and hyper-methylated underexpressed genes in CRC tissues and cell lines. Then, these candidate biomarkers in CRC cell lines and clinical serum samples including CRC, colorectal adenoma (CA) patients and healthy controls, were detected and the relationship with clinicopathologic parameters and their value as CRC diagnostic markers were analyzed.

## MATERIALS AND METHODS

### Bioinformatics analysis

mRNA data of 395 CRC patients were downloaded from the TCGA database. The “limma” package was used to calculate the DEGs between CRC tissues and normal colorectal tissues, and the filter was applied according to the thresholds |log_2_FC|>1 and *P* value <0.01. Specifically overexpressed or underexpressed genes in CRC tissues were verified by GEPIA. Overexpressed genes in CRC cell lines were selected by CCLE. Genes that encoded secretory proteins were screened according to HPA. The methylation status in CRC tissues and the CpG island locations of candidate genes were checked by UALCAN, MEXPRESS and UCSC.

### Clinical specimens

Serum and tissue samples were obtained from the First People’s Hospital of Yunnan Province and the Third People’s Hospital of Yunnan Province with informed consent, comprising a training cohort (60 CRC, 30 colorectal CA and 33 healthy controls) and a validation cohort (60 CRC, 30 CA and 30 healthy controls). The diagnosis of CRC was verified by endoscopy and pathological biopsy. None of the patients had received prior radiotherapy, chemotherapy or surgery treatment when blood samples were collected. In addition, 1 placental sample was used as a control to test the methylation status of *HPDL* and *CLIP4*.

### Cell culture and treatment

Seven human CRC cell lines (HT29, HCT116, SW480, SW620, RKO, DLD-1 and LOVO) and one normal colon cell line (CCD841CON) were obtained from the cell bank of the Chinese Academy of Sciences (Shanghai, China). All of cell lines were cultured in DMEM medium containing 10% fetal bovine serum (BI) and 100 IU/ml penicillin and streptomycin (Gibco) and maintained in 37°C in a humidified incubator with 5% CO_2_. For de-methylation treatment, cultured cells were incubated with 10 μm 5-aza-2’-deoxycytidine (Sigma, USA) for 3 days with medium changed every day.

### Quantitative PCR (Q-PCR) and Real-time PCR (RT-PCR)

The mRNA expression of candidate genes was analyzed by Q-PCR and RT-PCR. Total RNA was extracted with a Tissue Total RNA Isolation Kit (TSINGKE, China) and cDNA was obtained with a PrimerScript™ RT Reagent Kit (TSINGKE, China). Q-PCR was performed with 2 × Taq PCR Master Mix (TIANGEN, China). Real-time PCR was performed with EvaGreen 2 × qPCR MasterMix (Takara, Japan) in a CFX96TM Real-Time PCR System (BioRad, USA). The PCR reaction conditions were listed as follows: pre-denaturation at 94°C for 1.5 min, 30 cycles of predenaturation at 94°C for 10 s, annealing at 60°C for 20 s, extension at 72°C of 30 s, and ultimate extension at 72°C of 1 min. Primer sequences (10 μM concentration), annealing temperatures, and product sizes are listed in Table 1. The expression of the assayed genes was normalized to *GAPDH*.

**Table 1:** Primer sequences and product length.

**Gene**	**Primer sequence (5′–3′)**		**Annealing tem**	**Amplification size (bp)**
**Quantitative PCR**				
ADHFE1	F:GTGAGAGTGGAACCAACGGATTC	R:AGCAGCCTTACAGGTGTCCATG	60	120
ASCL2	F:CGCCTACTCGTCGGACGACAG	R:GCCGCTCGCTCGGCTTCCG	60	140
B3GNT3	F:AGGCACAGACTCACGGAGACAT	R:GTTGAGCACGAAGCTGGCGTTG	60	128
CCL24	F:TGAGAACCGAGTGGTCAGCTAC	R:TTCTGCTTGGCGTCCAGGTTCT	60	153
CDX1	F:GAGAAGGAGTTTCATTACAGCCG	R:GTTCACTTTGCGCTCCTTTGCC	60	132
CDX2	F:ACAGTCGCTACATCACCATCCG	R:CCTCTCCTTTGCTCTGCGGTTC	60	102
CEACAM5	F:GCCTCAATAGGACCACAGTCAC	R:CAGGTTAAGGCTACAGCATCCTC	60	115
CHRDL1	F:GGCTCTTTCAGAATCGGCAACC	R:AGAGACTGGGAAGGCACAGGTT	60	113
CLIP4	F:CTGTGAAGTGCCTCTTGGAGCA	R:GCTTGATTTCCTTAGCAGTGGCT	60	141
CPXM2	F:CAGAGGATCGACAGAATGTCCC	R:CATCCAGGCTATGACTGCTCTG	60	119
CST1	F:TGTGCCTTCCATGAACAGCCAG	R:CTGGCACAGATCCCTAGGATTC	60	130
CYP2S1	F:GATGGACGGTTCAGGAAGCATG	R:GGAGAAGGCTTGTAGGATGGTG	60	126
DEFA5	F:CTCCAGGAAAGAGCTGATGAGG	R:TCGGCAATAGCAGGTGGCTCTT	60	141
DEFA6	F:ATGACCAGGACTTTGCCGTCTC	R:CATGACAGTGCAGGTCCCATAG	60	140
EPCAM	F:GCCAGTGTACTTCAGTTGGTGC	R:CCCTTCAGGTTTTGCTCTTCTCC	60	122
EPHB2	F:CGCCATCTATGTCTTCCAGGTG	R:GATGAGTGGCAACTTCTCCTGG	60	130
FERMT1	F:CCAACTCTATGAGCAAGCCAGG	R:CCTGTGTTTCAGCAGACAACGAC	60	128
GFRA1	F:CATAGACTCCAGTAGCCTCAGTG	R:GTCACATCGGAGCCATTGCCAA	60	153
GMDS	F:TGAGTTCCTGCTGGAGAAAGGC	R:CAAGGCAGGTACTGTCAGTGAG	60	161
GSTM2	F:AGATCACCCAGAGCAACGCCAT	R:GGCTGTCCATAAACTGGTTCTCC	60	117
HPDL	F:AGCCAGGAAAGGAGAGGCAGAT	R:GGACTTGGTGAAGACCTGAAGC	60	119
IHH	F:GGACGCTATGAAGGCAAGATCG	R:CAGCGAGTTCAGGCGGTCCTT	60	150
KCNE3	F:GCCGTGATGACAACTCCTACATG	R:CACTACGCTTGTCCACTTTGCG	60	114
KRT8	F:ACAAGGTAGAGCTGGAGTCTCG	R:AGCACCACAGATGTGTCCGAGA	60	121
KRT18	F:GCTGGAAGATGGCGAGGACTTT	R:TGGTCTCAGACACCACTTTGCC	60	119
KRT20	F:CTGAGGTTCAACTAACGGAGCTG	R:AACAGCGACTGGAGGTTGGCTA	60	151
LGALS4	F:GGAACAGCCTTCTGAATGGCTC	R:CCATTGGCGTAAACCTTGAAGCG	60	130
LGR5	F:CCTGCTTGACTTTGAGGAAGACC	R:CCAGCCATCAAGCAGGTGTTCA	60	100
MUC3A	F:TCTTACACCTCGACTCCCGT	R:TTGGGGACGTGGTTGTATGG	60	262
MUC5B	F:CTGCTACGACAAGGACGGAAAC	R:AAGGCTGTGAGCGCACTGGATG	60	112
MUC13	F:TGGCTGTAACCAGACTGCGGAT	R:GCATCAGGACACTTGAGACTGG	60	123
NFE2L3	F:CCAGTTGCTTTCATCACAGCCTG	R:CACATCCTGACTTATAGCCTGGC	60	142
OLFM4	F:GACCAAGCTGAAAGAGTGTGAGG	R:CCTCTCCAGTTGAGCTGAACCA	60	138
PDLIM4	F:TGATGACAGCAAGGCTCAGGCA	R:AGGCTTGGTCTGCCATCTTCTG	60	123
PRR15	F:CCTGACACCTATGCCCAAACAG	R:CGTCCTGAGTTGGAGACCTTGA	60	146
SLC12A2	F:CCTCTACACAAGCCCTGACTTAC	R:CGTGAGTTTGGAGCACCTGTCA	60	124
SPINK4	F:TGCCAGTGGCAGCAGGAAAGC	R:CCAAGCAGAGCTGGCATTCATTC	60	144
SRMS	F:CCTCCTCAGAAGATGAACGACC	R:GGATGGACTTCTCCTCCGTCTA	60	197
UCHL1	F:CAGTTCAGAGGACACCCTGCTG	R:CCACAGAGCATTAGGCTGCCTT	60	122
GAPDH	F:GTCTCCTCTGACTTCAACAGCG	R:ACCACCCTGTTGCTGTAGCCAA	60	131
**Methylation-specific PCR**				
HPDL	F:ATTAGTTTAGGATTGAGAGTTTCGA	R:GACGAACACGTAAAAAACGAT	60	137
	F:ATTAGTTTAGGATTGAGAGTTTTGA	R:CTACCCAACAAACACATAAAAAACA	56	143
CLIP4	F:AGACGGGTAAGATTAGGTTTTCG	R:ACTAACAACGTCTACGAAATATCGC	60	173
	F:AAGATGGGTAAGATTAGGTTTTTG	R:CTAACAACATCTACAAAATATCACA	58	173

Abbreviations: F: forward primer; R: reverse primer; M: methylation; U: unmethylation; bps: base pairs.

### Enzyme-linked immunosorbent assay (ELISA)

Commercial ELISA kits were used to measure CEA, CK18, CK20, MUC13, CK8 and EPCAM (CUSABIO, China). Experiments were performed according to the manufacturers’ instructions. Optical density (OD) values were read at a wavelength of 450 nm using a 96-well microplate. All determinations were performed in duplicate.

### DNA extraction and bisulfite conversion

Genomic DNA from tissues and cells was extracted by using a TIANamp Genomic DNA Kit (TIANGEN, China). Genomic DNA from serum samples was extracted by the Axy Prep Body Fluid Viral DNA/RNA Miniprep Kit (Axy Prep, China). Complete bisulfite conversion of GC-rich DNA was performed by using the EZ DNA Methylation-Gold™. Kit (Zymo Research, USA).

### Methylation-specific PCR (MSP)

The methylation status of *HPDL* and *CLIP4* was detected by methylation-specific PCR assay utilizing the abovementioned bisulfite-modified DNA as templates, according to the previously mentioned protocols [19]. The methylated and de-methylated specific primer sequences (10 μM concentration), annealing temperatures, and product sizes are listed in Table 1. PCR products were evaluated by electrophoresis on ethidium bromide (EB)-stained 2% agarose gels. The sample was considered de-methylated *HPDL* when only a visible band was detected in un-methylation primer allele. The sample was considered hyper-methylated *CLIP4* when a visible band was detected in the methylation primer allele. All of the samples were amplified twice to check the accuracy of the results.

### Statistical analysis

The differences in CEA, CK18, CK20, MUC13, CK8 and EPCAM among the study groups were compared via nonparametric analysis. The correlations between CK18, CK20, de-methylated *HPDL*, hyper-methylated *CLIP4* and clinicopathologic parameters were evaluated by the chi-square test or Fisher’s exact test. To evaluate the validity of each studied parameter, sensitivity and specificity were used. All statistical analyses were performed using SPSS 19.0 (SPSS Inc., USA).

## RESULTS

### High levels of serum CK18 and CK20 were detected in CRC and CA patients

**Figure 1:**
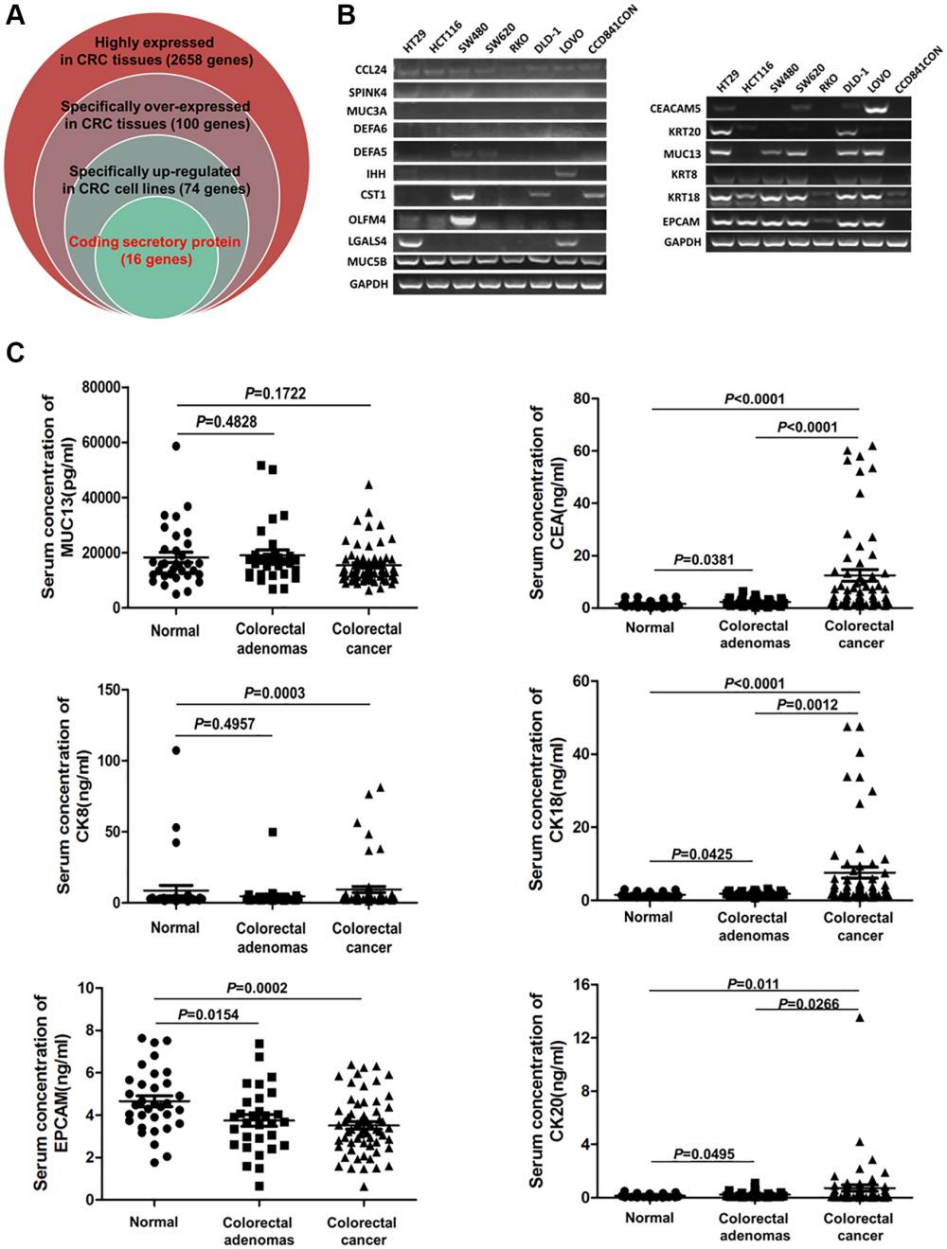
**The serum levels of CEA, CK18, CK20, CK8, MUC13 and EPCAM in CRC, CA patients and healthy controls.** (**A**) Screening specific genes that encode secretory proteins in CRC by bioinformatics. (**B**) Testing the expression of candidate serum biomarker genes by Q-PCR in 8 cell lines. (**C**) Detection of the serum levels of CEA, CK18, CK20, CK8, MUC13 and EPCAM in CRC, CA patients and healthy controls by ELISA.

**Figure 2:**
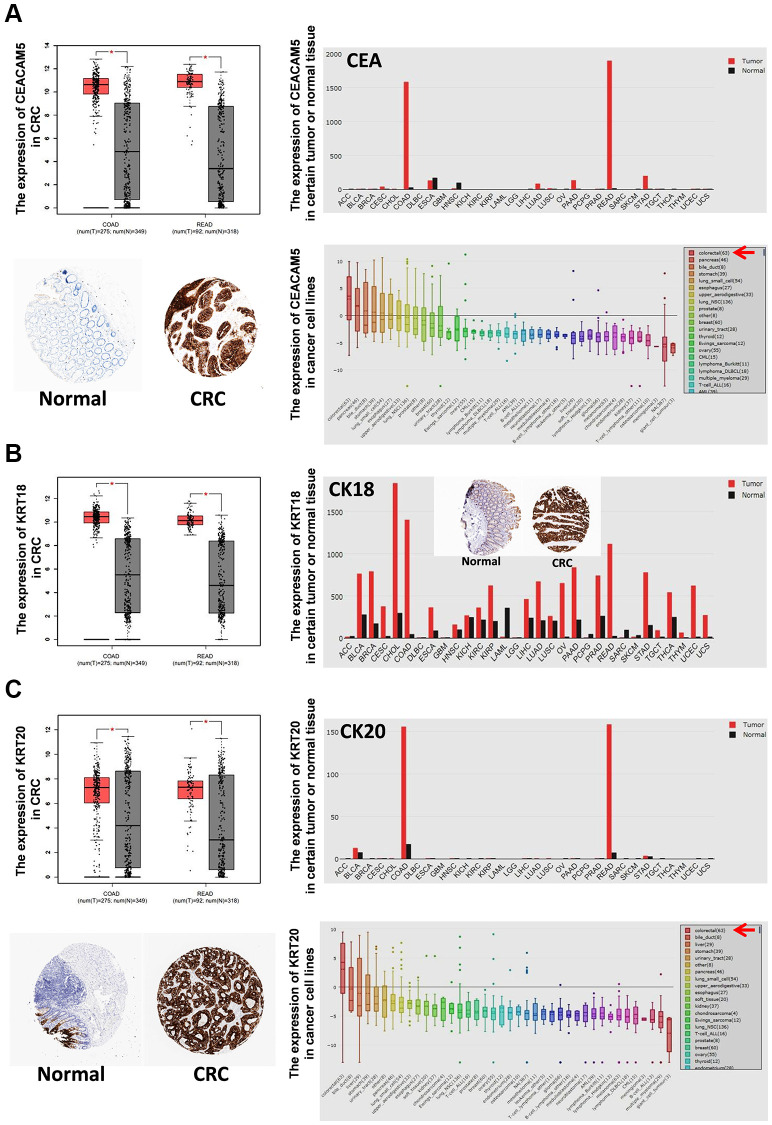
**The expression of CEA, CK18 and CK20 in tumor tissues and cancer cell lines.** The mRNA expression of CEA (**A**), CK18 (**B**) and CK20 (**C**) in certain tumor tissues and CRC tissues was analyzed by GEPIA. The protein expression of CEA (**A**), CK18 (**B**) and CK20 (**C**) in CRC tissues was stained by immunohistochemistry (IHC) and analyzed by HPA. The mRNA expression of CEA (**A**), CK18 (**B**) and CK20 (**C**) in cancer cell lines was analyzed by CCLE. Abbreviations: COAD: colon adenocarcinoma; READ: rectum adenocarcinoma.

TCGA and GTEx analysis revealed 2658 genes highly expressed in CRC tissues (log_2_-fold >1, *P* < 0.01). Compared with human normal tissues, 100 genes were specifically overexpressed in CRC tissues (fold log_2_ >3, *P* < 0.01) (Supplementary Table 1). Among them, 74 genes were overexpressed in CRC cell lines (rank Top 3) (Supplementary Table 2), and 16 genes encoded secretory proteins (Supplementary Table 3; Figure 1A). Then, Q-PCR was used to detect the expression of 16 genes in 7 CRC cell lines (HT29, HCT116, SW480, SW620, RKO, DLD-1, LOVO) and 1 normal colon cell line (CCD841CON). It was found that *CEACAM5*, *KRT8*, *KRT18*, *KRT20*, *MUC13* and *EPCAM* were significantly overexpressed in CRC cell lines (Figure 1B). With ELISAs to test serum CEA (encoded by *CEACAM5*), CK8 (encoded by *KRT8*), CK18 (encoded by *KRT18*), CK20 (encoded by *KRT20*), MUC13 and EPCAM in CRC, CA patients and healthy controls, the results showed that CEA, CK18 and CK20 were significantly higher in CRC and CA patients than in healthy controls (all *P* < 0.05) (Figure 1C). GEPIA, HPA and CCLE also verified CEA, CK18 and CK20 overexpressed in CRC tissue; CEA and CK20 specifically increased in CRC cell lines (Figure 2).

### De-methylated *HPDL* was observed in CRC and CA serum

**Figure 3:**
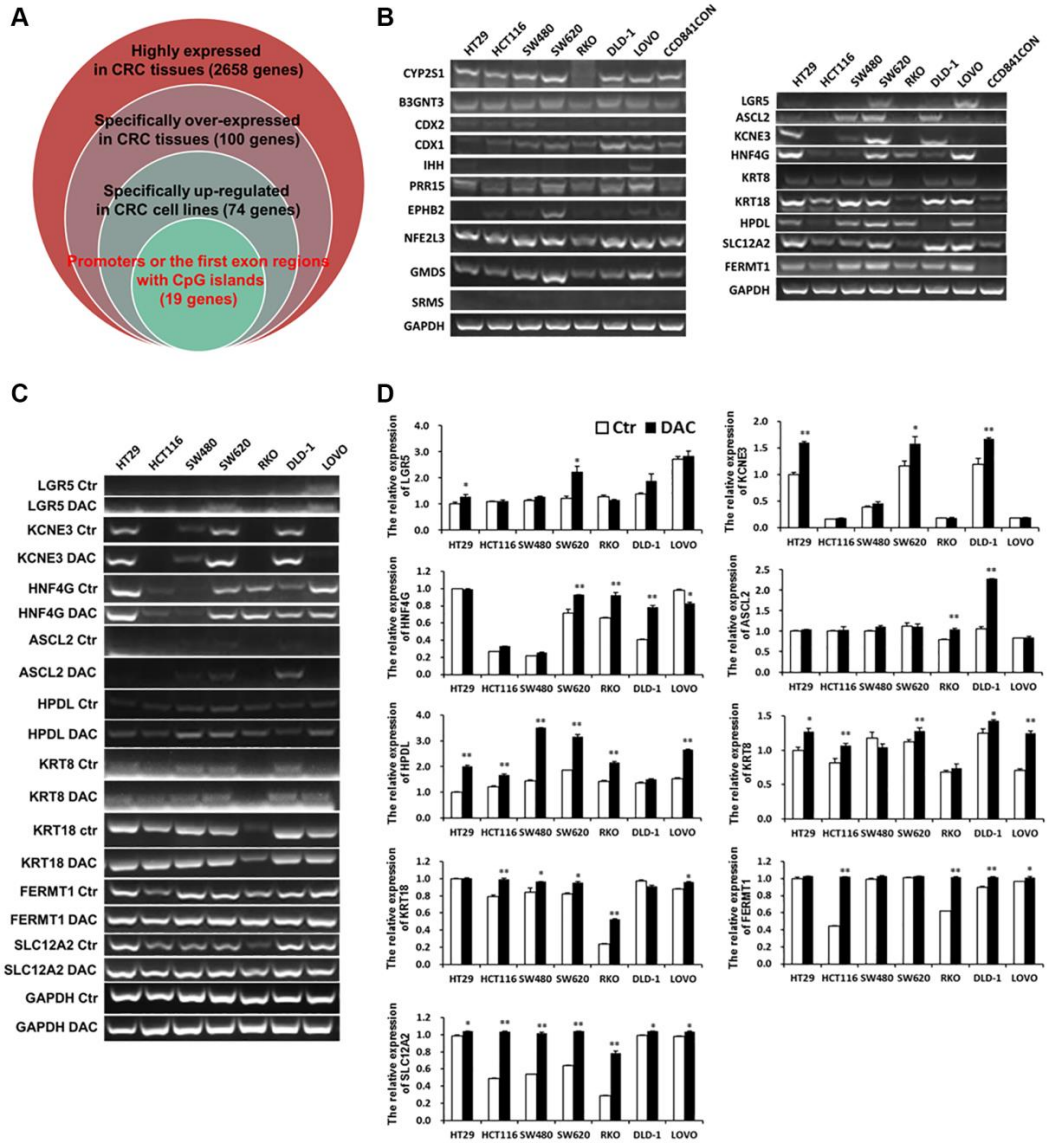
**Specific overexpressed and de-methylated genes in CRC tissues were screened by bioinformatics and verified in CRC cell lines by Q-PCR or RT-PCR.** (**A**) Screening specific overexpressed and de-methylated genes in CRC tissues by bioinformatics. (**B**) Testing the expression of de-methylated genes by Q-PCR in CRC cell lines and a normal colon cell line. The expression of de-methylated genes was tested using Q-PCR (**C**) and RT-PCR (**D**) in CRC cell lines after treatment with DAC.

**Figure 4:**
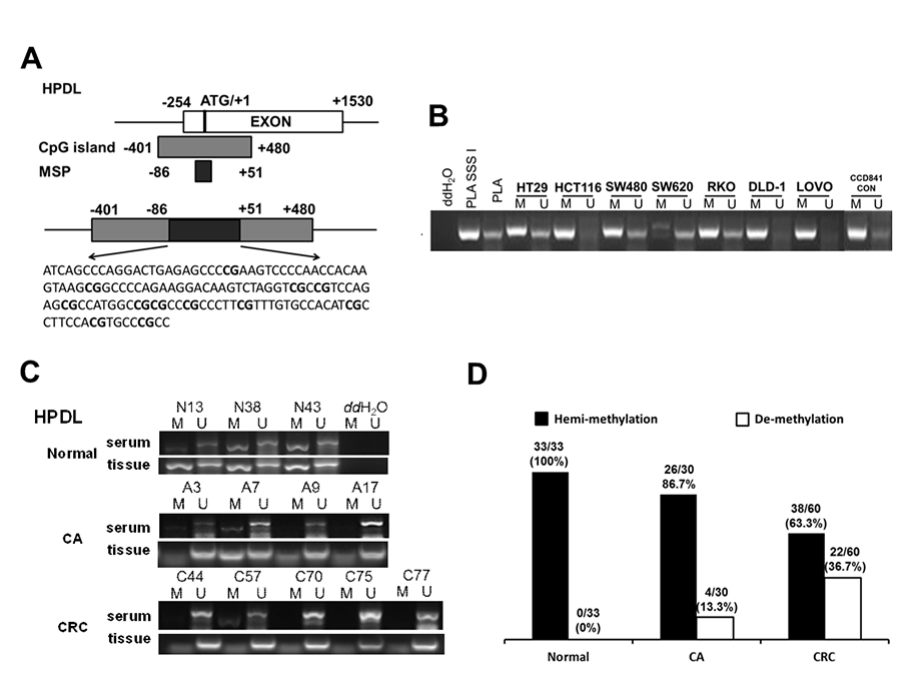
**Serum de-methylated *HPDL* in CRC, CA patients and healthy controls.** (**A**) Schematic illustration of the gene structure of *HPDL*, the CpG island region and the position of MSP primers. (**B**) Detected *HPDL* methylation status in 8 cell lines. Placental DNA (or treated by SSSI) represented a positive control of de-methylated or methylated status. Abbreviations: M: methylation; U: un-methylation. (**C**) Representative serum and tissue methylation status of *HPDL* in CRC, CA patients and healthy controls. (**D**) Frequency of serum *HPDL* methylation status in 60 CRC, 30 CA patients and 33 healthy controls.

**Figure 5:**
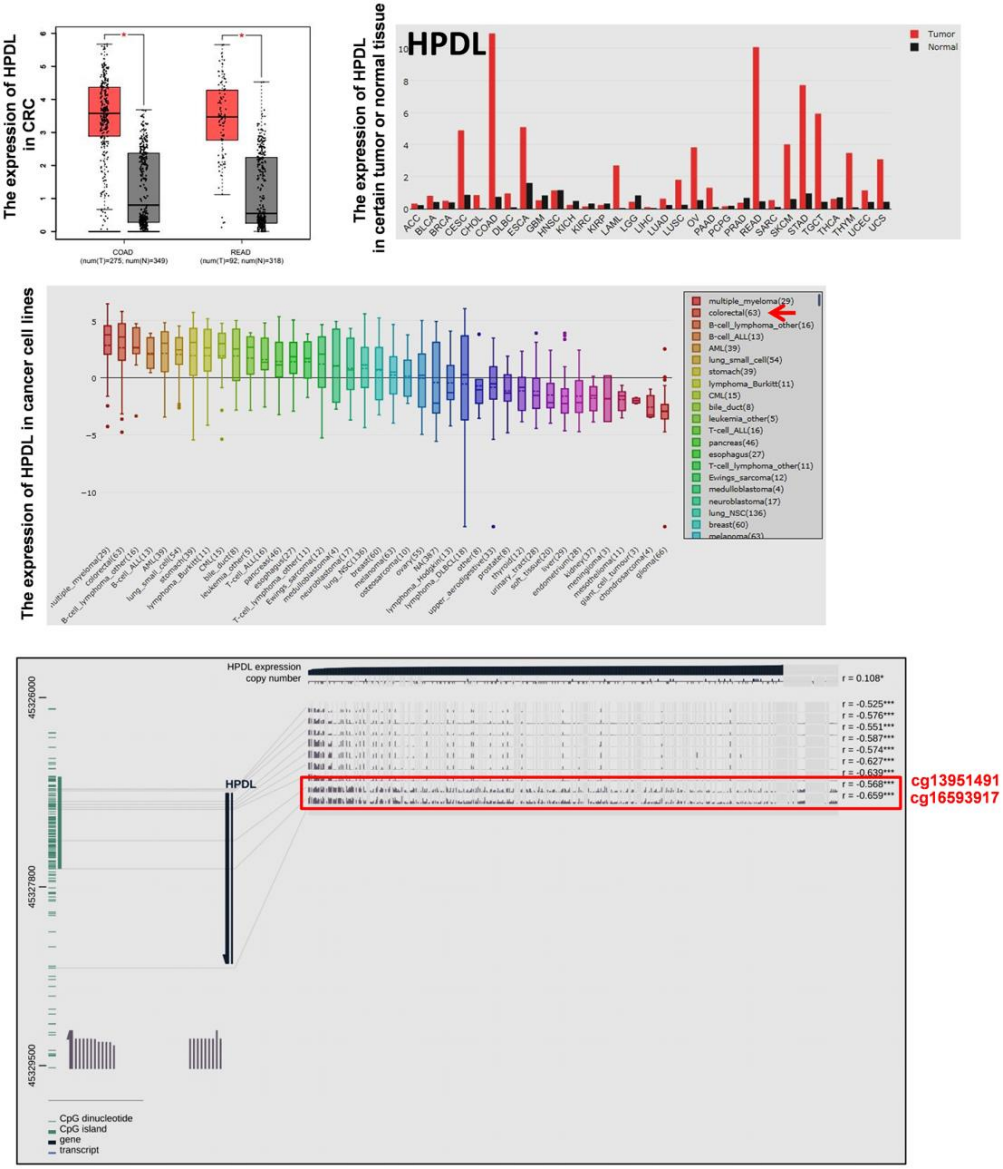
**The expression of *HPDL* in tumor tissues and cancer cell lines and the methylation status of *HPDL* in CRC tissues.** The mRNA expression of *HPDL* in certain tumor tissues and CRC tissues was analyzed by GEPIA. The mRNA expression of *HPDL* in cancer cell lines was analyzed by CCLE. The relationship between the expression and promoter methylation level of *HPDL* in CRC tissues was analyzed by MEXPRESS. The red frame showed *HPDL* methylation status in CRC and normal tissues (Probes ID: cg13951491 and cg16593917). Abbreviations: COAD: colon adenocarcinoma; READ: rectum adenocarcinoma.

Normally, DNA de-methylation can lead to genome instability and high expression of oncogenes. Based on the previous bioinformatics analysis results, among 74 specifically overexpressed genes, UCSC showed that 19 genes possessed CpG islands in their promoters or the first exon region (Supplementary Table 4; Figure 3A). Detecting the expression of 19 genes in 7 CRC cell lines and CCD841CON revealed that *HPDL*, *LGR5*, *ASCL2*, *KCNE3*, *HNF4G*, *KRT8*, *KRT18*, *SLC12A2* and *FERMT1* were significantly overexpressed in CRC cell lines (Figure 3B). To test the relationship between DNA methylation status and the expression of these genes in CRC, the expression of 9 genes in 7 CRC cell lines treated with 5′-aza-2′-deoxycytiding (DAC) were detected. As shown in Figure 3C and 3D, *HPDL*, *KRT8*, *KRT18*, *FERMT1* and *SLC12A2* were increased in CRC cell lines in response to DAC treatment. According to the CpG island region, MSP primers for these genes were designed and the methylation status of 5 genes in CRC cell lines and a normal colon cell line were tested. The results revealed that only *HPDL* presented more de-methylation status in CRC cell lines (especially SW620) than CCD841CON (Figure 4A and 4B). GEPIA and CCLE demonstrated that *HPDL* was highly expressed in CRC tissues and CRC cell lines; MEXPRESS also revealed that CRC tissues possessed *HPDL* de-methylated regions (Probes ID: cg13951491 and cg16593917) compared with normal tissue (Figure 5). These results indicated that *HPDL* overexpressed in CRC may be upregulated by DNA de-methylation.

Because serum contains a certain amount of DNA derived from lysed tumor cells, the methylation status of *HPDL* was detected in the serum of 60 CRC patients, 30 CA patients and 33 healthy controls. As shown in Figure 4C, *HPDL* de-methylation was detectable in CRC and CA patients but not in healthy controls. Statistical analysis showed that the de-methylated frequency of serum *HPDL* was 36.7% (22/60) in CRC patients and 13.3% (4/30) in CA patients (Figure 4D). Additionally, representative cases consisting of 20 CRC, 10 CA patients and 10 healthy controls were selected to detect *HPDL* methylation status in serum and colorectal normal or tumor tissue from the same patient. The results indicated that the *HPDL* methylation status in serum was almost consistent with that in CRC tissues (Figure 4C).

### Hyper-methylated *CLIP4* was identified in CRC and CA serum

**Figure 6:**
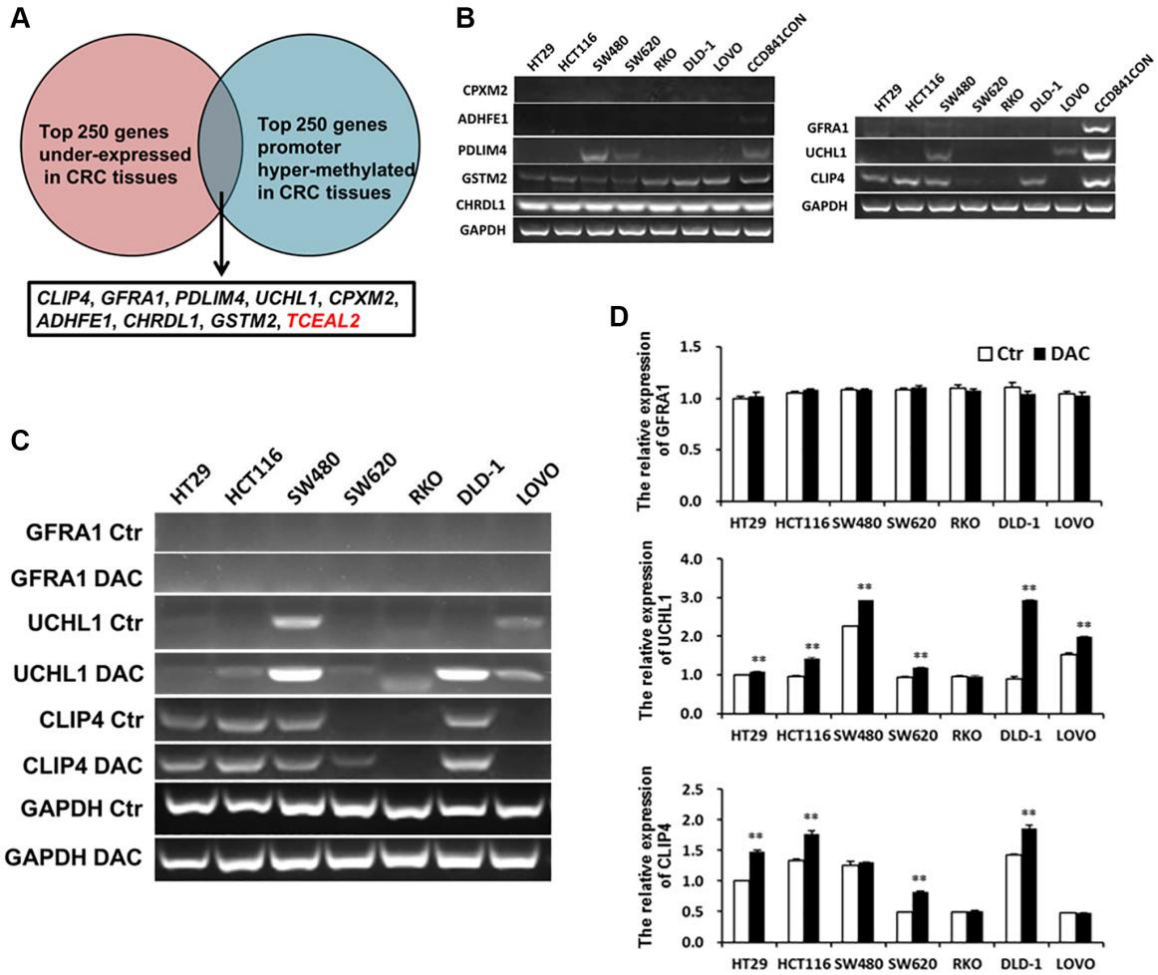
**Underexpressed and hyper-methylated genes were screened in CRC tissues by bioinformatics and verified in CRC cell lines by Q-PCR or RT-PCR.** (**A**) Screening underexpressed and hyper-methylated genes in CRC tissues by bioinformatics. (**B**) Testing the expression of hyper-methylated genes by Q-PCR in CRC cell lines and a normal colon cell line. The expression of hyper-methylated genes was tested using Q-PCR (**C**) and RT-PCR (**D**) in CRC cell lines after treatment with DAC.

**Figure 7:**
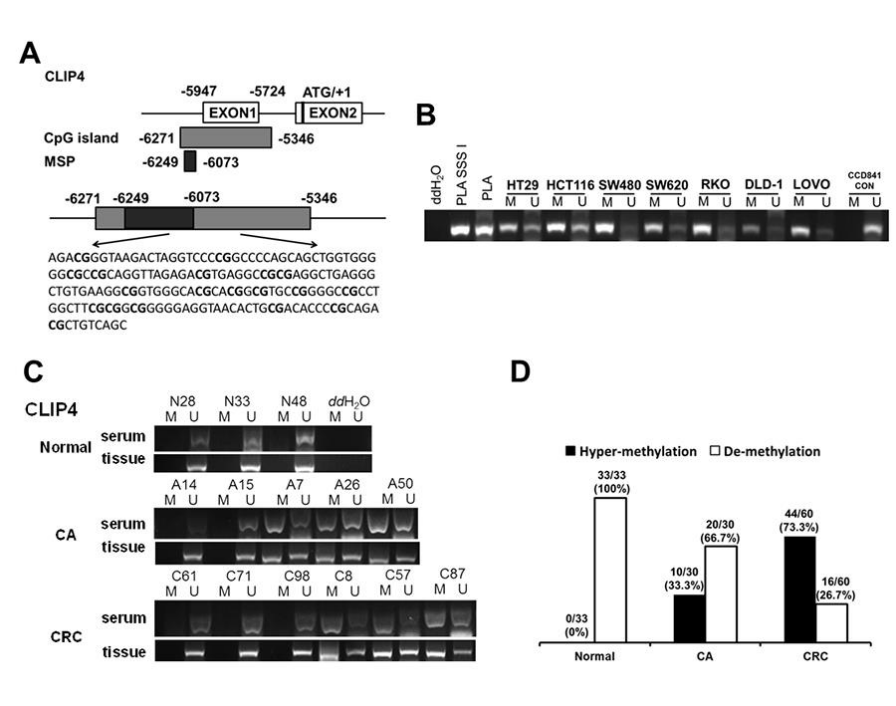
**The serum hyper-methylated status of *CLIP4* in CRC, CA patients and healthy controls.** (**A**) Schematic illustration of the gene structure of *CLIP4*, the position of CpG islands and MSP primers. (**B**) Detecting *CLIP4* methylation status in 8 cell lines. Placental DNA (or treated by SSSI) represented a positive control for de-methylation or methylation. Abbreviations: M: methylation; U: un-methylation. (**C**) Representative serum and tissue methylation status of *CLIP4* in CRC, CA patients and healthy controls. (**D**) Frequency of serum *CLIP4* methylation status in 60 CRC, 30 CA patients and 33 healthy controls.

**Figure 8:**
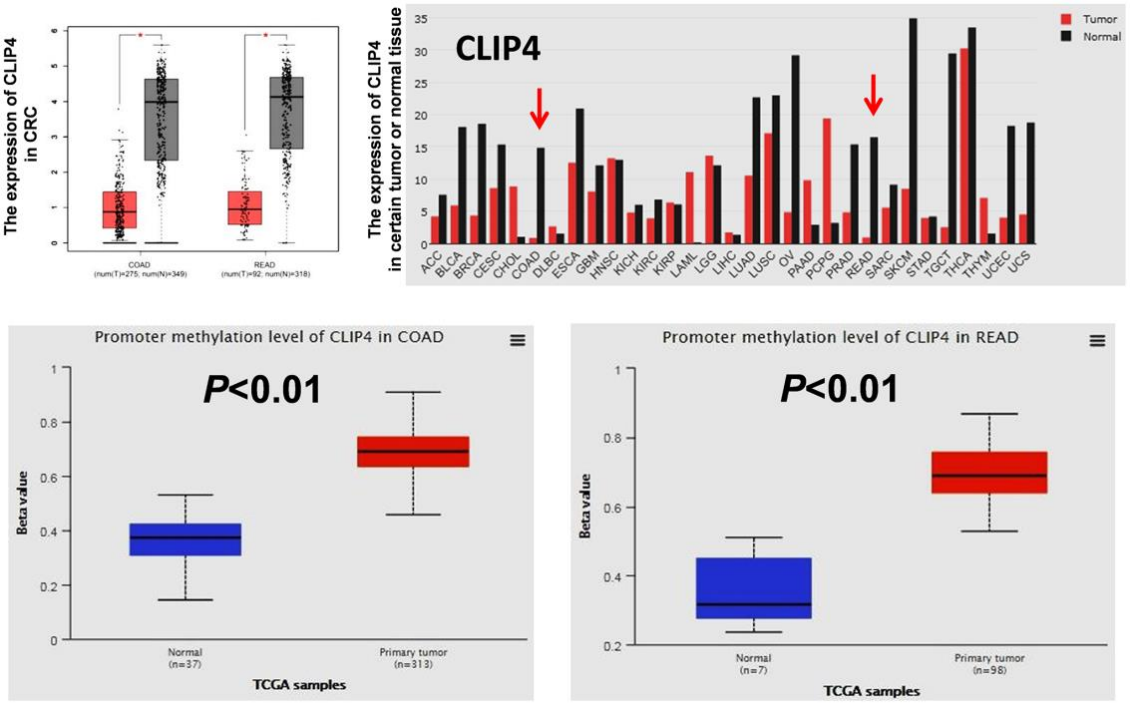
**The expression of *CLIP4* in tumor tissues and cancer cell lines and the methylation status of *CLIP4* in CRC tissues.** The mRNA expression of *CLIP4* in certain tumor tissues and CRC tissues was analyzed by GEPIA. The methylation level of *CLIP4* in CRC and normal tissues was analyzed by UALCAN. Abbreviations: COAD: colon adenocarcinoma; READ: rectum adenocarcinoma.

DNA hyper-methylation is associated with tumor suppressor gene silencing and defects in cell cycle regulation, resulting in tumor development and progression. Comparing the top 250 underexpressed genes in TCGA (Supplementary Table 5) with the top 250 promoter hyper-methylated genes in UALCAN (Supplementary Table 6), we found that 9 genes showed underexpression and promoter hyper-methylation in CRC tissue. UCSC exhibited that 8 genes possessed CpG islands located in promoters (Figure 6A). Detecting the expression of 8 genes in 7 CRC cell lines and CCD841CON revealed that *CLIP4*, *GARA1* and *UCHL1* were underexpressed in CRC cell lines and overexpressed in a normal colon cell line (Figure 6B). As determined by Q-PCR and RT-PCR, after DAC treatment, *CLIP4* and *UCHL1* were upregulated in CRC cell lines (Figure 6C and 6D). According to the CpG islands located in the promoter, MSP primers were designed and tested the methylation status of 2 genes in CRC cell lines and a normal colon cell line. The results showed that *CLIP4* presented significant hyper-methylation in CRC cell lines and total de-methylation in a normal colon cell line (Figure 7A and 7B). GEPIA and UALCAN also indicated that *CLIP4* was underexpressed and hyper-methylated in CRC tissue (Figure 8). By detecting the methylation status of *CLIP4* in serum from 30 CRC, 20 CA patients and 33 healthy controls, it was found that *CLIP4* hyper-methylation was detectable in CRC and CA but not in healthy serum (Figure 7C). By statistical analysis, the hyper-methylation frequency of serum *CLIP4* was 73.3% (44/60) in CRC and 33.3% (10/30) in CA patients (Figure 7D). Furthermore, representative cases consisting of 20 CRC patients, 10 CA patients and 10 healthy controls were chosen to detect the *CLIP4* methylation status in serum and colorectal normal or tumor tissue from the same patient. The results illustrated that the *CLIP4* methylation status in serum was completely consistent with that in CRC tissue (Figure 7C).

### Clinical values of serum CK18 and CK20 and de-methylated *HPDL* and hyper-methylated *CLIP4* for CRC diagnosis

A training cohort consisting of 60 patients with CRC (age range: 46–87 years), 30 patients with CA (age range: 26–77 years), and 33 healthy controls (age range: 33–75 years), and a validation cohort consisting of 60 CRC (age range: 43–88 years), 30 CA (age range: 35–82 years), and 30 healthy controls (age range: 31–72 years) were enrolled in this study. The baseline and clinical characteristics of the patients and controls are summarized in Table 2.

**Table 2:** Demographic and clinicopathologic characteristics of clinical cohorts.

**Characteristics**	**Training cohort (*N* = 123)**			**Validation cohort (*N* = 120)**		
	**Normal**	**CA**	**CRC**	**Normal**	**CA**	**CRC**
Age						
≤50	7 (21.2%)	5 (16.7%)	9 (15.0%)	14 (46.7%)	8 (26.7%)	7 (11.7%)
>50	26 (78.8%)	25 (83.3%)	51 (85.0%)	16 (53.3%)	22 (73.3%)	53 (88.3%)
Sex						
Male	13 (39.4%)	19 (63.3%)	39 (65.0%)	17 (56.7%)	23 (76.7%)	41 (68.3%)
Female	20 (60.6%)	11 (36.3%)	21 (35.0%)	13 (43.3%)	7 (23.3%)	19 (31.7%)
Tumor location						
Colon			22 (36.7%)			19 (33.9%)
Rectum			38 (63.3%)			37 (66.1%)
Tumor Size (cm)						
≤4			30 (50.0%)			22 (39.3%)
>4			30 (50.0%)			34 (60.7%)
TNM stage						
I + II			34 (56.7%)			16 (28.6%)
III + IV			26 (23.3%)			40 (71.4%)
Differentiation						
Well			7 (11.7%)			5 (8.9%)
Moderate			48 (80%)			42 (75.0%)
Poor			5 (8.3%)			9 (16.1%)
Lymphovascular invasion						
Absent			36 (60.0%)			21 (37.5%)
Present			24 (40.0%)			35 (62.5%)
CEA						
<5 ng/ml			28 (46.6%)			21 (35%)
≥5 ng/ml			32 (53.3%)			39 (65%)
CA199						
<37 U/ml			46 (76.7%)			38 (73.1%)
≥37 U/ml			14 (23.3%)			14 (26.9%)
CA125						
<35 U/ml			54 (90.0%)			48 (92.3%)
≥35 U/ml			6 (10.0%)			4 (7.7%)

Abbreviations: CA: colorectal adenomas; CRC: colorectal cancer.

The relationships between CK18, CK20 or *HPDL*, *CLIP4* methylation status and various clinicopathologic parameters in CRC patients are summarized in Table 3. According to the results, in the training cohort, CK18 was significantly correlated with TNM stage, differentiation grade, CEA and CA19-9 (all *P* < 0.05). CK20 was closely correlated with tumor size and CA199 (*P* < 0.05). De-methylated *HPDL* was apparently associated with tumor size, CEA and CA199 (*P* < 0.05). Hyper-methylated *CLIP4* was markedly associated with differentiation grade and CEA (*P* < 0.05) in CRC patients. In the validation cohort, CK20 was significantly correlated with tumor location and CA199 (*P* < 0.05). Hyper-methylated *CLIP4* was closely associated with age, TNM stage, differentiation grade, lymphovascular invasion and CEA (all *P* < 0.05).

**Table 3:** Correlation of serum biomarkers level or methylation status with clinicopathological characteristics.

**Characteristics**	**Training cohort**								**Validation cohort**			
	**CK18**		**CK20**		***HPDL***		***CLIP4***		**CK20**		***CLIP4***	
	**Negative**	**Positive**	**Negative**	**Positive**	**Hemi-methylated**	**Un-methylated**	**Hyper-methylated**	**De-methylated**	**Negative**	**Positive**	**Hyper-methylated**	**De-methylated**
Age												
≤50	5	4	8	1	7	2	6	3	3	4	3	4
>50	27	24	36	16	31	20	38	13	35	18	45	8
*P* value	0.588		0.205		0.281		0.449		0.405		**0.025**	
Sex												
Male	20	19	27	12	25	14	29	10	27	14	34	7
Female	12	9	16	5	13	8	15	6	11	8	14	5
*P* value	0.436		0.399		0.542		0.518		0.376		0.493	
Tumor location												
Colon	11	11	13	9	12	10	13	9	8	11	15	4
Rectum	21	17	30	8	26	12	31	7	27	10	31	6
*P* value	0.45		0.09		0.212		0.057		**0.04**		0.72	
Tumor Size (cm)												
≤4	18	12	25	5	24	6	22	8	15	7	16	6
>4	14	16	18	12	14	16	22	8	20	14	30	4
*P* value	0.219		**0.042**		**0.007**		0.614		0.577		0.167	
TNM stage												
I + II	22	12	24	10	22	12	22	12	8	8	6	10
III + IV	10	16	19	7	16	10	22	4	27	13	40	0
*P* value	**0.039**		0.533		0.506		0.074		0.24		**0.000**	
Differentiation												
Well	6	1	6	1	7	0	2	5	3	2	1	4
Moderate	26	22	35	13	30	18	37	11	28	14	36	6
Poor	0	5	2	3	1	4	5	0	4	5	9	0
*P* value	**0.013**		0.203		0.017		**0.009**		0.407		**0.001**	
Lymphovascular invasion												
Absent	22	14	25	11	24	12	24	12	13	8	11	10
Present	10	14	18	6	14	10	20	4	22	13	35	0
*P* value	0.112		0.434		0.35		0.128		0.582		**0.000**	
CEA												
<5 ng/ml	27	1	22	6	24	4	17	11	12	9	9	12
≥5 ng/ml	5	27	21	11	14	18	27	5	26	13	39	0
*P* value	**0.000**		0.206		**0.001**		**0.038**		0.325		**0.000**	
CA199												
<37 U/ml	30	16	36	10	33	13	32	14	29	9	29	9
≥37 U/ml	2	12	7	7	5	9	12	2	3	11	14	0
*P* value	**0.001**		**0.046**		**0.018**		0.2		**0.001**		0.092	
CA125												
<35 U/ml	31	23	40	14	33	21	40	14	31	17	39	9
≥35 U/ml	1	5	3	3	5	1	4	2	1	3	4	0
*P* value	0.07		0.216		0.276		0.512		0.285		0.456	

*P* < 0.05 is considered statistically significant.

Further analysis suggested that under the best cutoff values defined by the tertiles method, in the training cohort, CK18 detected CRC with 46.67% sensitivity and 87.3% specificity; CK20 with 28.33% sensitivity and 90.47% specificity; de-methylated *HPDL* with 36.67% sensitivity and 93.65% specificity; and hyper-methylated *CLIP4* with 73.33% sensitivity and 84.13% specificity. Random combined analysis suggested CK20/hyper-methylated *CLIP4* with 91.67% sensitivity and 82.54% specificity. In the validation cohort, CK20 detected CRC with 36.7% sensitivity and 88.3% specificity; hyper-methylated *CLIP4* with 80% sensitivity and 85% specificity; and CK20/hyper-methylated *CLIP4* with 95% sensitivity and 81.7% specificity (Table 4). Considering sensitivity and specificity, CK20/hyper-methylated *CLIP4* was a potential diagnostic biomarker for CRC.

**Table 4:** Evaluation of serum biomarkers level or methylation status in detection of CRC.

**Markers**	**Training cohort**		**Validation cohort**		**Best cut-off value**
	**Sensitivity**	**Specificity**	**Sensitivity**	**Specificity**	
CEA	53.33%	85.71%	65.00%	83.30%	≥5 ng/ml
CA199	23.33%	92.06%	26.90%	91.70%	≥37 U/ml
CK18	46.67%	87.30%			≥3 ng/ml
CK20	28.33%	90.47%	36.70%	88.30%	≥0.5 ng/ml
*HPDL*	36.67%	93.65%			De-methylated
*CLIP4*	73.33%	84.13%	80.00%	85.00%	Hyper-Methylated
CEA or *CLIP4*	81.67%	73.02%			
CK18 or *CLIP4*	80.00%	77.78%			
CK20 or *CLIP4*	91.67%	82.54%	95.00%	81.70%	
HPDL or *CLIP4*	81.67%	80.95%			
CK20 or *HPDL or CLIP4*	93.33%	76.19%			

## DISCUSSION

Cytokeratin is a conserved group of proteins that form the cytoplasmic structure of epithelial cells and tissues. Cytokeratin 20 (CK20) is a type 1 cytokeratin. It is a prominent component of the intestinal epithelium. CK20 expression is confined to astrointestinal epithelium, urothelium, and Merkel cells of the epidermis, as well as malignancies that originate from the aforementioned sites [20]. According to previous studies, Y Imai indicated that CK20 expression in tumor tissues was an independent prognostic factor of poorly differentiated adenocarcinoma of the colon and rectum [21]. As one of the most investigated markers for the detection of circulating CRC cells, CK20 mRNA in serum is widely tested by RT-PCR for predicting recurrence and poor prognosis of CRC [22–29]. However, the efficacy of CK20 protein in serum as a biomarker for early CRC screening and diagnosis is not clear. In this study, we offered a precise value of serum CK20 protein in CRC diagnosis with 28.33% sensitivity and 90.47% specificity in the training cohort and 36.7% sensitivity and 88.3% specificity in the validation cohort. We also detected that CK20 presented higher levels in CA patients with a rate of 16.67% in the training cohort. This result indicated that CK20 possessed diagnostic potential for early CRC screening.

*CLIP4*, as a member of the CAP-Gly domain containing linker protein (CLIP) family, which is involved in plus-end binding of microtubules, has been implicated in immune response-related biological processes, cell migration and viability in certain cancer metastases [30]. Hyper-methylation of *CLIP4* has been shown diagnostic potential for CRC in serum [31]. S.O. Jensen reported that hyper-methylated *CLIP4* was capable of distinguishing serum from CRC patients and healthy controls (the area under the curve was 0.88) [32]. By testing the methylation status in CRC serum, we found that serum hyper-methylated *CLIP4* detected CRC with a sensitivity of 73.33% and specificity of 84.13% in the training cohort and 80% sensitivity and 85% specificity in the validation cohort. We also detected hyper-methylated *CLIP4* in CA patients at a rate of 33.3% but not in healthy controls. This implied that serum *CLIP4* hyper-methylation could be used for early CRC screening.

Due to the highly heterogeneous nature of CRC, a single tumor marker is unlikely to become a stand-alone diagnostic test as the commonly insufficient sensitivity and/or specificity. Using a panel of tumor markers and testing with different methods for CRC diagnosis has the potential to be an effective approach. With systematic bioinformatics screening and clinical verification, our study showed that a combination of serum CK20 and hyper-methylated *CLIP4* was a novel and effective biomarker for CRC diagnosis with 91.67% sensitivity and 82.54% specificity in the training cohort; and 95% sensitivity and 81.7% specificity in the validation cohort. It was more sensitive than *CLIP4* hyper-methylated alone in stool specimens (90.3% sensitive, 88.4% specificity) [33]. Comparing with previous serum CRC biomarkers, CK20/hyper-methylated *CLIP4* was more effective than CEA/MMP-7/TIMP-1 (sensitivity: 70.3%, specificity: 91.3%) [34], RUNX3/SFRP1/CEA (sensitivity 84.71%) [35], LRG1/EGFR/ITIH4/ HPX/SOD3 (sensitivity: over 70%, specificity: 89%) [36], anti-SLP2/-p53/-SEC61B/-PLSCR1 (sensitivity: 64.1%, specificity: 80%) [37], miR-203a-3p/miR-145-5p/miR-375-3p/miR-200c-3p (sensitivity: 81.52%, specificity: 73.33%) [38], miR-144-3p/miR-425-5p/miR-1260b (sensitivity: 93.8%, specificity: 91.3%) [39], and less than CCL20/IL-17A (sensitivity: 96.1%, specificity: 96.5%) [40]. Elevated CCL20 and IL-17A levels may reflect inflammatory condition, which can increase the false-positive fraction (FPF) of CRC detection [40]. In comparison, CRC cells overexpressed CK20 and showed hyper-methylated *CLIP4*. Serum CK20/hyper-methylated *CLIP4* represented the tumor status of patients. The combination of serum CK20/hyper-methylated *CLIP4* could decrease FPF of CRC detection.

In this study, we found several limitations, which should be regarded as preliminary research, and upcoming surveys should focus on several issues. First, CRCs can be characterized by their primary tumor location. Left-sided colon cancer (LCC), including rectum and right-sided colon cancer (RCC), is different in pathogeneses, molecular characteristics, incidences and prognoses. In LCC, chromosomal instability has been detected in approximately 75% more than 30% of RCCs [41]. With increased chromosomal instability, LCC has been associated with more frequent overexpression of the epidermal growth factor receptor (EGFR) ligands, *EGFR*, *EREG*, *AREG*, *HERS*, *VEGF-1* and *COX-2* [42]. In RCC, Hypermutation is more prevalent. RCC has been shown to be associated with an increase in *RAS* and phosphoinositide 3-kinase pathway mutations, *BRAF* mutations, and *TGFβR2* mutations. CpG island methylator phenotype (CIMP)-high and microsatellite-high subtype (MSI) have also been detected in RCC [43]. According to our study, in the validation cohort, elevated levels of CK20 were significantly correlated with the tumor location of the colon, not the rectum. Therefore, whether the expression of CK20 in tumor tissues and the serum level of CK20 are different between LCC and RCC and whether serum CK20 could distinguish LCC from RCC need to be further studied. Second, serum CK20 mRNA is a biomarker of circulating CRC cells. Serum CK20 protein originates from circulating CRC cells or CRC tumor tissue, which urgently needs to be determined. Therefore, for serum CK20 protein-positive patients, serum CK20 mRNA should be detected, and CK20 protein in CRC tumor tissues should be examined by IHC. Third, bioinformatics and DNA methylomics showed that breast and gastric cancer tissues presented hyper-methylated *CLIP4* [44–46]. The diagnostic value of hyper-methylated *CLIP4* in serum for breast cancer and gastric cancer has not yet been reported. Thus, a study involving several cancer types should be conducted to verify the specificity of hyper-methylated *CLIP4* and CK20/hyper-methylated *CLIP4* for CRC diagnosis. Fourth, through clinical serum sample validation, we found that only the combination of CK20 and hyper-methylated *CLIP4* displayed high sensitivity and specificity for CRC diagnosis. The reason is unclear. Therefore, the biological function of CK20 and CLIP4 in CRC and the relationship between them should be further explored. In addition, our study was performed on a limited number of CRC individuals (Only 120 patients were enrolled) from two centers. In the future, a study involving several hospitals/clinics from different regions covering a large population should be conducted to avoid overestimation of the sensitivity and specificity of serum CK20/hyper-methylated *CLIP4*. Finally, although none of the CRC patients had received radiotherapy, chemotherapy or surgery treatment prior to blood collection, they had already been clinically diagnosed by endoscopy and pathological biopsy. Serum biomarkers would be more likely detectable in clinical patients than subclinical patients. Therefore, a large number of blood samples from a health examination center should be collected and serum CK20/hyper-methylated *CLIP4* should be detected. Then, for patients positive for serum CK20 or hyper-methylated *CLIP4* should be examined by endoscopy and pathological biopsy to verify the ability of serum CK20/hyper-methylated *CLIP4* to diagnose CRC.

## CONCLUSIONS

In summary, from systematical bioinformatics screening to clinical serum sample validation, this study shows that the combination of serum CK20 and hyper-methylated *CLIP4* is a novel effective biomarker for CRC diagnosis.

## Supplementary Materials

Supplementary Tables
