# Supplementary material for: Coupling of serum CK20 and hyper-methylated CLIP4 as promising biomarker for colorectal cancer diagnosis: from bioinformatics screening to clinical validation
Source: Aging (Albany NY). 2021 Dec 29;13(24):26161–79. doi: 10.18632/aging.203804 (PMC8751608; doi:10.18632/aging.203804)
Supplement: Supplementary Tables [file aging-13-203804-s001.pdf]

SUPPLEMENTARY TABLES

Supplementary Table 1. 100 genes specifically overexpressed in CRC tissue.

CRC vs. Colon and Rectal normal tissue (log_2 fold >1, *P* value <0.01) and CRC vs. Different part of normal tissues (log_2 fold >3, *P* value <0.01)
CEACAM5 GPX2 RNF43 KRT18 UBD CST1 CCL20 ASCL2 FERMT1 NOX1 OLFM4 CYP2S1 EPCAM VIL1 MMP1 B3GNT3 IGHG4 CCL24 MISP MUC13 SPINK4 CDX2 LY6G6D CDX1 CDH17 IHH RP11-357H14.17 ATP10B MMP12 TSPAN8 PRR15 C6orf223 KRT8 UBE2SP2 MUC3A EPHB2 GUCY2C PLS1 NFE2L3 LGR5 CTD-2377D24.6 COL10A1 GMDS AP000349.2 KRT20 C021218.2 GPA33 PPP1R14D ACSL5 MEP1A LINC01559 BCL2L15 RP11-150O12.3 C6orf222 GALNT4 HPDL TRPM2-AS RP5-881L22.5 SLC12A2 AP003774.1 CASC9 KCNE3 HNF4G C2CD4A TRIM15 APOBEC1 FAM111B LINC01207 GPR35 EPSTI1 LINC00483 RP11-304L19.1 GALS4 RP11-474D1.3 ABHD11-AS1 EFA6 CALML4 AP000439.3 CASC21 EPS8L3 AC005355.2 HTR1D FUT4 AL3ST2 TMEM211 HOXB-AS4 UC5B CXCR3 RP11-234B24.2 DEFA5 ATF2 KRT8P11 FEZF1-AS1 RP11-187E13.1 RN7SKP54 POU5F1B SRMS AC016735.1 TLX1 XFP4

Supplementary Table 2. 74 genes specifically overexpressed in CRC cell lines.

CRC vs. Colon and Rectal normal tissue (log_2 fold >1, *P* value <0.01) and CRC vs. Different part of normal tissues (log_2 fold >3, *P* value <0.01) and Over expression in CRC cell lines (Rank top 3)
CEACAM5 GPX2 RNF43 KRT18 CST1 ASCL2 FERMT1 NOX1 OLFM4 CYP2S1 VIL1 B3GNT3 CCL24 MISP MUC13 SPINK4 CDX2 CDX1 CDH17 IHH RP11-357H14.17 ATP10B TSPAN8 PRR15 C6orf223 KRT8 MUC3A EPHB2 GUCY2C PLS1 NFE2L3 LGR5 CTD-2377D24.6 GMDS KRT20 AC021218.2 GPA33 PPP1R14D ACSL5 MEP1A BCL2L15 RP11-150O12.3 C6orf222 HPDL RP5-881L22.5 SLC12A2 KCNE3 HNF4G TRIM15 APOBEC1 GPR35 LINC00483 LGALS4 RP11-474D1.3 DEFA6 CALML4 AP000439.3 EPS8L3 AC005355.2 GAL3ST2 TMEM211 HOXB-AS4 MUC5B RP11-234B24.2 DEFA5 BATF2 KRT8P11 RP11-187E13.1 RN7SKP54 POU5F1B SRMS AC016735.1 TLX1 RXFP4

Supplementary Table 3. 16 CRC specifical overexpressed genes which encoding secreted proteins.

CRC vs. Colon and Rectal normal tissue (log_2 fold >1, *P* value <0.01) and CRC vs. Different part of normal tissues (log_2 fold >3, *P* value <0.01) and Over expression in CRC cell lines (Rank top 3) and Secreted proteins
KRT18 CST1 OLFM4 EPCAM CCL24 SPINK4 IHH KRT8 MUC3A DEFA6 MUC5B DEFA5 CEACAM5 MUC13 KRT20 LGALS4

Supplementary Table 4. 19 CRC specifical overexpressed genes possessed CpG islands in their promoters or the first exon region.

CRC vs. Colon and Rectal normal tissue (log_2 fold >1, *P* value <0.01) and CRC vs. Different part of normal tissues (log_2 fold >3, *P* value <0.01) and Over expression in CRC cell lines (Rank top 3) and CpG islands in promoters or the first exon region
KRT18 CYP2S1 B3GNT3 CDX2 CDX1 IHH PRR15 KRT8 EPHB2 NFE2L3 GMDS SRMS ASCL2 FERMT1 LGR5 HPDL SLC12A2 KCNE3 HNF4G

Supplementary Table 5. Top 250 underexpressed genes in CRC tissue.

CRC vs. Colon and Rectal normal tissue (log_2 fold <−1, *P* value <0.01)
TMBIM4 ARHGEF17 COL6A2 GFRA1 TXLNGY MAOB MBNL1-AS1 CES1 PODN GOLGA8A ACACB CA4 HMCN2 SFRP1 ITGA5 FBXL22 SLC22A17 CILP PHYHD1 PCSK1N CLDN5 CLIP4 IL11RA GREM2 GABBR1 PTGS1 MAP6 CLMP REEP1 ZEB1 GPRASP1 RP11-6O2.3 STON1 MRVI1 ZG16 WISP2 CHGA EPHA7 C15orf52 SCGN APOD MFAP4 EBF4 MIR4697HG PALM MEG3 CSPG4 STARD9 TNS2 PDLIM4 PRRT2 ABI3BP DDR2 BHMT2 AKAP12 NKX2-3 PHLDB2 OSR1 AC002398.12 COL6A1 MEIS2 LINC01573 TGFB1I1 KDM5D GSTM1 TSPYL2 CCDC136 CAV1 S100B ARHGEF26 APBB1 PTGIS FGFR1 MYOCD CSRP1 RERG DPP6 CLIP3 RP11-166D19.1 MAGI2-AS3 UCHL1 CALD1 ANK2 PYGM DNAJB5 ACKR1 TMOD1 USP32P1 PDZD4 PER1 PDLIM7 GRIK5 L1CAM DTNA PLEKHO1 AGTR1 RGS2 SOX10 RASGRP2 PDLIM3 KANK2 ZBTB16 SVIL FERMT2 CFL2 PPP1R1A MST1L VIP RIC3 FENDRR ACTA2-AS1 BOC DACT3 CADM3 TMEM35 NPTX1 ADAMTS9-AS1 DTX3 ATP2B4 C8orf88 PRUNE2 CHRM2 NEXN BCHE PDZRN4 PDE2A FAM46B CLU SFRP5 RIMKLB PCP4 FAM107A PPP1R12B GPM6A MSRB3 SDPR LYNX1 CPXM2 SMTN SLC26A10 C20orf166-AS1 ADCY5 OGN RP11-286H15.1 ABCA8 SLC2A4 ADHFE1 SPARCL1 CFD RAMP1 FLNA LDB3 LYVE1 SCARA5 CCDC69 MAL PDK4 RPS4Y1 UBXN10-AS1 FAM129A MEIS1 NNAT TNXB SCN7A KCNMA1 RGMA SRPX AP000892.6 THBS4 NCAM1 CCL14 CHRDL1 SGCA AOC3 PRPH ASB2 MGP TPM2 MASP1 SORBS1 MRGPRF MYLK AL442127.1 GNAO1 JPH2 C2orf40 FXYD6 CASQ2 PLXNB3 CACNA1H GSTM2 MAMDC2 ANGPTL1 FLNC FABP4 PLP1 TAGLN CA1 MORN5 KCNMB1 ARHGEF25 TNS1 CORO6 ATP1A2 PRIMA1 MIR143HG RNA5SP216 HIF3A PLN TCEAL2 CRYAB LGI4 PRELP LMO3 ITGA7 PPP1R14A GPX3 HSPB7 POPDC2 LIMS2 MAB21L2 MYL9 FXYD1 PLIN4 PGM5 HAND1 PNCK RBFOX3 HAND2-AS1 PGM5-AS1 TACR2 C7 FHL1 DPT RBPMS2 HAND2 HSPB8 PSD SYNPO2 SPEG LMOD1 ADAM33 HSPB6 CNN1 RP11-394O4.5 ADH1B SYNM ACTG2 MYH11 DES

Supplementary Table 6. Top 250 promoter hyper-methylated genes in CRC tissue.

CRC vs. Colon and Rectal normal tissue (Beta value: 0.7–0.5, *P* value <0.01)
VWC2 DPP10 EFCC1 KHDRBS2 FGF5 ADHFE1 PTPRT FAIM2 EYA4 DOK5 PREX2 GATA5 CMTM3 COL4A1 COL4A2 GFRA1 SNAP91 DPY19L2 CMTM2 DKFZP434H168 SYT9 RALYL FBN2 FAM19A4 SPATA32 INA ITGA8 ADAMTS5 FIGN PRDM14 CNKSR2 RAB6C IRX4 GALNT13 CFAP100 MARCH11 NALCN DYDC1 DYDC2 ST8SIA5 CHST10 LOC283392 TRHDE GPR26 ZNF582 BARHL2 MIR129-2 OTX2-AS1 LRFN5 PCDH10 EID3 PTPN5 FGF12 FOXE1 MIR137 MIR124-3 DOK6 CPXM2 MMP23B SORCS3 FNDC1 CRHR2 QRFPR GALNTL6 GSC2 GRIA4 SLC13A5 TMEM132C NPBWR1 NR2E1 CCDC178 GRWD1 CHL1 SOX17 CHRDL1 ST6GALNAC5 SLC16A12 COL26A1 RYR2 WDR86 SALL1 PCDH8 FGF8 GLRB GRIK3 SORCS1 MMP16 GCM2 PTF1A DMRT3 OPRK1 UNC80 DBX2 LAMA1 DRD4 TFPI2 SDC2 SIM1 CIDEA ADGRA1 COL23A1 PDLIM4 VIPR2 CCAR2___BRINP1 SLITRK4 ZNF814 VAV3 ZNF132 LOC157627 ESX1 MKX SLC6A3 HCN1 PLPPR4 SHISA9 BHLHE23 CLDN10 NPY GABBR2 VSX1 ZNF75A SH3GL3 NR0B1 ADAM12 FOX12 GPR101 TCEAL2 FOXD2 FOXD2-AS1 NRK MYOD1 ZNF549 ADRA1A VSTM2B C1QL2 CFAP46 ITIH5 PAX7 PHOX2A SALL3 OLIG2 LINC00472 ZNF334 HOXA5 POU4F2 MSC FUT9 GAD2 EFEMP1 FAM19A5 HNF4G MDFIC NXPH2 SHISA2 EFS DNAH11 LDOC1 TCERG1L GRIA2 ADCY4 ZNF85 SLC18A3 SLC4A11 SOX3 SLC32A1 HOXA2 TWIST1 TRPC6 NID1 FGF3 ZNF667 FAM162B BTG4 ZNF415 NID2 CLIP4 LOC100188949 GPR83 SLC6A2 CDO1 GJD2 NELL1 MIR348 TEME196 CRISPLD1 ADCYAP1 UTF1 PCDH17 CUX2 VEGFC STX1B HOXD10 SFRP2 GHSR CYP7B1 PTH2R RXRG SLC6A15 ADAMTS16 EXOC3L2 NEFH NPTX2 SIX6 SULT4A1 TMEM108 TBX20 ANKRD20A8P GPR27 UCHL1 FBN1 GALR2 TBX5 DRD5 ZFP42 JAM3 TLX3 ZNF542P AJAP1 SYNDIG1 CCNA1 NCAM2 PCDHGC3 KDR WNK3 ADCY8 DGKI PDPN EML1 HS3ST3A1 FREM3 DTX1 EVC2 CDH3 KCNG1 FBXO39 NGF ACSS3 TMEM132D CLVS2 HTR1A TDRD10 SHE TMEM26 GRM7 CCDC181 GSTM2 STAC FAM218A SLC6A11 USP44
